# “Liking You Doesn’t Mean I Want Your Dickpic”: (Cyber)Rape Culture Predicts Women’s Perception and Emotional Responses to Unsolicited Genital Images

**DOI:** 10.1007/s10508-026-03463-9

**Published:** 2026-06-11

**Authors:** Rocío Vizcaíno-Cuenca, Hugo Carretero-Dios, Mónica Romero-Sánchez

**Affiliations:** 1 Department of Research Methods in Behavioral Sciences, Mind, Brain and Behavior Research Center (CIMCYC), Faculty of Psychology, University of Granada, Campus de Cartuja s/n, 18071 Granada, Spain; 2 Department of Social Psychology, Brain and Behavior Research Center (CIMCYC), University of Granada, Granada, Spain

**Keywords:** Cybersexual violence, Dickpic, Unsolicited genital images, Emotions, Rape culture, Cyberflashing

## Abstract

The unsolicited receipt of genital images is a widespread form of cyber-sexual violence against women. While many women describe these experiences as humiliating or disgusting, others perceive them as harmless or even flattering. Building on a qualitative pilot study (92 women participants), we investigated how women evaluate and emotionally react to unsolicited genital images, and how these responses are influenced by prior sexual context and myths about cyber-sexual violence. A total of 218 Spanish women participants reported their acceptance of myths about cyber-sexual violence and evaluated a hypothetical incident of receiving an unsolicited genital image with the sexual context manipulated in a between-participants design. Results showed that women evaluated the incidents less positively and exhibited more anxiety, anger-hostility, and sadness, and less happiness and fewer feelings of power after exposure to both incidents of unsolicited genital images. Importantly, women with a higher acceptance of myths about cyber-sexual violence evaluated the incidents more positively, which in turn was associated with more positive and fewer negative feelings when the woman previously showed sexual interest toward the perpetrator (vs. no sexual interest or the control condition). These findings highlight the need for interventions that challenge these myths, empowering women to resist pressures to normalize such behaviors.

## Introduction

“He asked if I fancied meeting for coffee and a walk by the river and suggested they swap phone numbers to make arrangements easier. Almost instantly, he sent me closeups of his penis. I felt totally shocked. Nothing in the conversation had made me think he was going to do that. I felt really worried because it seemed like a deception, I was confused, I didn’t know what he was really looking for, I didn’t feel safe”— Leah (Sarner, 2019)

Experiences like the one shared by Leah in an interview with *The Guardian*, in which women receive unsolicited genital images, are increasingly common worldwide. While unsolicited genital images are also reported in the everyday use of hookup apps among same-sex attracted men—with 5% to 48% of non-heterosexual men reporting having received such images (Oswald et al., 2020; Smith, 2018)—evidence suggests that around 90% of recipients are women (United Nations [UN] Women, 2020). In the United States and the United Kingdom, half of the adult women surveyed had received unsolicited genital images (Pew Research Center, 2017; Smith, 2018; UN Women, 2021), while in Spain, our research context, between 20 and 75% of women aged 18 to 65 reported experiencing these incidents, which made them feel humiliated, offended, or intimidated (Delegación del Gobierno contra la Violencia de Género, 2020; Rodríguez-Domínguez et al., 2026).

Research has shown the harmful long-term consequences of unsolicited genital images, including anxiety, depression, body shame, and low self-esteem (Iroegbu et al., 2024), as well as eating disorders, alcohol abuse (Oliver et al., 2024), and social isolation (Champion et al., 2022; Henry et al., 2020). In offline contexts, studies have not only documented these types of consequences following sexual violence, but have also shown that individual attitudes—such as the acceptance of rape myths—can shape how such incidents are interpreted and how intensely emotional reactions are experienced (see Bohner et al., 2023 for a review). This suggests that emotional responses are directly associated with the violence itself, but can also be influenced by cognitive frameworks used to make sense of it.

While research has provided valuable insight into emotional responses to sexual violence in offline settings, much less is known about these processes in online contexts. Despite the growing prevalence of behaviors like unsolicited genital image sharing, little is known about how women emotionally respond to such incidents—let alone whether attitudes such as the endorsement of myths about cyber-sexual violence influence their emotional reactions or evaluations of the incident. These questions are the focus of the present research.

### The Unsolicited Genital Image: Conceptualization, Motivations, and Justification

Social networks are said to have transformed interpersonal communication through enabling instant exchanges of text and images across physical and geographical boundaries (Lieberman & Schroeder, 2020). These interactions can involve sexual content that is either consensual or non-consensual (Bonilla et al., 2021; Jeacock et al., 2025; Paasonen et al., 2019; Thorne et al., 2024). Consensual exchanges, such as sexting or sharing sexual images, are generally reported to be experienced positively (Mori et al., 2020; Thorne et al., 2024). In contrast, non-consensual exchanges—such as the unsolicited sending of genital images—are widely recognized as a form of cyber-sexual violence, because they constitute unwanted or non-consensual sexually aggressive behaviors carried out through digital technologies, primarily targeting women (Henry & Powell, 2015), and are generally reported as negative by recipients (Champion et al., 2022; Hayes & Dragiewicz, 2018; Marcotte et al., 2021Powell & Henry, 2019).

Specifically, unsolicited genital images—also referred to as cyberflashing—occur when men send photos of their genitals without consent. Unlike consensual sexual exchanges, this behavior is understood primarily as an exercise of misogyny, rather than sexual desire (Amundsen, 2021; Jeacock et al., 2025). Similar to street harassment, unsolicited genital images may function on two levels: in some instances, senders might present them as harmless flirtation or compliments; in others, they may be intended to assert control, reinforce gendered power dynamics, and remind women of their sexual objectification (Amundsen, 2021; Anciones-Anguita & Checa-Romero, 2024; Paasonen et al., 2019). This dual logic—of apparent seduction masking domination—has also been observed in empirical findings. For instance, Oswald et al. (2020) found that the most reported motivation for unsolicited genital images was to receive images in return or get sexual excitement; however, participants who reported sending these images also showed stronger endorsement of hostile and dominance-oriented attitudes against women (McArthur et al., 2025). Moreover, other studies have shown that the unsolicited sending of such images to women is closely linked to broader patterns of harassment, often occurring alongside negative social media commentary, revenge porn, and gender-based hate speech (Bonilla et al., 2021; Ringrose et al., 2021).

Furthermore, the unsolicited receipt of genital images is not reported as a rare or “aberrant” event, but rather an “ordinary” and insidious incident—one whose cumulative effects might be considered as damaging as those of physical violence (Hayes & Dragiewicz, 2018). Nevertheless, public discourse often appears to focus on more overt forms of violence, such as physical assault, thereby overlooking and trivializing other manifestations of discrimination and violence against women, including unsolicited genital images. For instance, Vizcaíno-Cuenca et al. (2024) found that women who reported receiving such images were frequently met with reactions of minimization—e.g., “Look, honey, I don’t know what toilet it came out of, but your statement is very stupid. I prefer a dick pic a thousand times over a rape or assault…”—and victim-blaming—e.g., “And why doesn’t she show the full conversation? Ayyy naughty!!! Maybe we should look at the context in which the photo was sent.”

In response to the reporting of these incidents, these comments may serve to distract from and undermine broader efforts to challenge dominant cultural values—such as rape culture—that normalize and legitimize this type of behavior (Hayes & Dragiewicz, 2018). In this regard, research in offline contexts has shown that such reactions are often rooted in a set of sexist beliefs or interpretive frameworks commonly referred to as myths (Bohner et al., 2023).

### Myths About Cyber-Sexual Violence

According to sexual script theory (Simon, 2017), individuals understand and interpret sexual behavior through culturally shared narratives and social interactions—these sexual scripts not only shape desire, but also guide how individuals perceive and respond to their own and their partner’s behavior. Ryan (2011) demonstrated that sexual scripts are influenced by rape culture, particularly rape myths, which lead individuals to interpret acts of offline sexual violence as desired and consensual encounters. However, in online settings, complex interactions emerge between digital architectures, symbolic discourses, and the collective activity of socially embedded users, reflecting broader societal narratives that differ from traditional rape myths (Dodge, 2016; Sharabi, 2021).

Research has highlighted the presence of macro-level cultural attitudes, beliefs, and online social norms that contribute to the normalization of cyber-sexual violence (McCaughey & Cermele, 2022; Vizcaíno-Cuenca et al., 2024). These shared cultural frameworks have been conceptualized as (cyber)rape culture, defined here as a societal-level phenomenon that legitimizes and trivializes sexual harm within digital environments. This process is sustained through discursive practices—such as victim-blaming, the trolling of survivors, and the dissemination of sexist memes—as well as deeply ingrained normative assumptions regarding gender and sexuality (Dodge, 2016; McCaughey & Cermele, 2022).

Importantly, while (cyber)rape culture operates at a structural and cultural level, recent research has shown that its core assumptions are also reflected at the individual level in the form of specific attitudinal beliefs, conceptualized as myths about cyber-sexual violence (Vizcaíno-Cuenca et al., 2024). These myths refer to a set of descriptive and prescriptive beliefs concerning the nature, causes, and consequences of cyber-sexual violence, as well as the roles and responsibilities attributed to victims and perpetrators (Vizcaíno-Cuenca et al., 2025). At the individual level, women’s attitudes may be associated with differences in the cognitive and emotional impact of cyber-sexual violence, potentially affecting how incidents such as the receipt of unsolicited sexual images are experienced (Abrams et al., 2003; Heider, 1958; Simon, 2017). Accordingly, greater endorsement of these myths has been associated with increased victim-blaming and the minimization of cyber-sexual violence experiences (Vizcaíno-Cuenca et al., 2025).

The literature has shown that rape myths negatively impact women victims of offline sexual violence, often fostering self-blame and reducing reporting rates due to a lack of acknowledgment and validation of their experiences (Brooker & Butler, 2021; Heath et al., 2011). In this context, rape victims who hold stronger rape myths reported worse physical and mental health (Bernstein et al., 2024), but also, they were less likely to recognize their experiences as violence (Wilson et al., 2018, 2021). These consequences appear to extend into online contexts as well, where women who experienced different forms of cyber-sexual violence frequently reported higher anxiety and depression, alongside lower self-esteem and body appreciation (Iroegbu et al., 2024)—effects that were especially pronounced among those who endorse myths about cyber-sexual violence (Vizcaíno-Cuenca et al., 2026). Similarly to offline sexual violence contexts, victims of cyber-sexual violence who endorse myths about cyber-sexual violence may experience significant emotional and psychological consequences. However, the lack of recognition and denial of these behaviors as violence may be amplified in more “ordinary” and commonplace forms of violence—such as unsolicited genital images—where behaviors are less readily recognized as violence, shaping victims’ emotional responses (Custers & Van den Bulck, 2013; Hayes & Dragiewicz, 2018; Kelly, 1987), yet still having an accumulative effect on health (Vizcaíno-Cuenca et al., 2026).

### Factors Affecting Women’s Evaluation and Reactions to Sexual Violence

Prior research has indicated that women typically react with negative emotions to the unsolicited receipt of genital images (Marcotte et al., 2021; Rodríguez-Domínguez et al., 2026). These reactions are consistent with broader emotional responses documented in the context of offline sexual violence, where anger-hostility and anxiety have been frequently reported (Calogero et al., 2021; Moya-Garófano et al., 2022). Anger-hostility tends to emerge when individuals perceive themselves as targets of demeaning offenses and clearly attribute blame to the perpetrator, often prompting a reactive or confrontational stance, whereas anxiety—more closely linked to the perception of threat rather than the offense itself—typically results in avoidant behavior (Giner-Sorolla & Russell, 2009; Lazarus, 1993). Moreover, sadness has also been commonly observed in response to such incidents (Calogero et al., 2021; Marcotte et al., 2021), reflecting states associated with pain, loss, and helplessness (Lazarus, 1993).

However, some studies have found that a minority of women also report ambivalent or even positive reactions to incidents of sexual violence (e.g., unsolicited genital images or street catcalls), such as feelings of happiness (Marcotte et al., 2021; Rodríguez-Domínguez et al., 2026) and power (Moya-Garófano et al., 2022). Both emotions reflect a highly positive evaluation of the situation; however, while happiness typically arises when individuals perceive progress toward a personal goal (Lazarus, 1993), feelings of power are associated with experiences of empowerment and increased self-esteem (Liss et al., 2011).

In this regard, in a qualitative study with young women, Ringrose et al. (2021) found that some perceived the receipt of unsolicited genital images as a sign of popularity and desirability. Another study indicated that women with more traditional conceptions of gender roles viewed offline sexual harassment as flattering, believed it was done with good intentions, or perceived it as merely a joke (Saunders et al., 2017). Similarly, Moya-Garófano et al. (2022) found that women who endorsed more sexist attitudes evaluated street harassment more positively, which, in turn, was associated with more positive and fewer negative emotions.

As described above—and similar to street harassment—unsolicited genital images are often sent by men as an expression of sexual interest (Amundsen, 2021; Paasonen et al., 2019), and, rather than being perceived as a form of domination, this behavior may be interpreted more positively by women who endorse traditional gender roles in heterosexual interactions (Liss et al., 2011) or who hold distorted beliefs about sexual dynamics (e.g., rape myths) (Ryan, 2011). Following this, it is possible that those women who endorse myths about cyber-sexual violence may feel flattered or even enjoy the sexual attention—particularly when the situation aligns with preexisting misinterpreted schemas (e.g., perceived sexual interest as a justification of unwanted sexual attention) about what constitutes implicit sexual consent (Newstrom et al., 2021), denying the violent or hostile nature of the perpetrator’s behavior. These interpretations suggest that individual beliefs and social norms may shape not only how such incidents are perceived but also how women emotionally respond to them—especially when contextual cues reinforce their preconceptions.

Therefore, women’s emotional reactions may be influenced not only by the incident itself but also by how they interpret and evaluate it through the lens of their beliefs and the specific situational context. While prior research has examined how sexist attitudes or adherence to traditional gender roles shape responses to offline sexual violence (e.g., Moya-Garófano et al., 2022; Saunders et al., 2017), little is known about how myths about cyber-sexual violence influence women’s evaluations and emotional reactions to unsolicited genital images—particularly when factors like the presence or absence of a sexual context are considered.

### Current Studies

Taken together, our research aimed to examine the influence of both situational and individual factors on women’s perceptions and emotional reactions to unsolicited genital images. In a pilot study, we collected information about individuals’ personal experiences and emotional responses to the unsolicited receipt of genital images. Subsequently, in an experimental study, we analyzed how the acceptance of myths about cyber-sexual violence influences perceptions and emotional reactions to a hypothetical scenario involving the unsolicited receipt of a genital image, in which the victim’s sexual interest was manipulated, along with a control condition.

## Study 1

### Pilot Study

Before examining how attitudes—such as adherence to myths about cyber-sexual violence—and contextual factors may influence women's emotional reactions to receiving unsolicited genital images, we first sought to develop a more detailed understanding of the phenomenon itself. Therefore, we conducted a pilot study with a sample similar to that of the main study to: (1) explore key situational features of these incidents, such as the online platforms where the images were received, the type of relationship with the sender, and the context in which the image was sent, and (2) identify the emotional responses most commonly reported by women after receiving unsolicited genital images.

This exploratory work aimed to replicate findings from prior research on the dynamics of unsolicited genital image sharing, while also gathering useful information to refine the manipulations and measures planned for the main study. At the same time, this pilot study seeks to ensure that our survey instrument accurately captures the emotional and contextual nuances reported by participants.

### Participants

A total of 92 women aged 18–46 years (*M* = 24.63, *SD* = 5.05) reported experiences of the unsolicited receipt of genital images. Among all women, 72.8% identified as heterosexual, 25% as bisexual, 1.1% as gay/lesbian and 1.1% as pansensual. In terms of educational background, women reported the following: 5.4% had completed vocational training, 31.5% had a general certificate of education, 58.7% held a university degree, and 4.3% had completed Ph.D. studies.

### Procedure and Measures

The sample was recruited through various online platforms, including X, Facebook, Instagram, Telegram, WhatsApp, and email. Participants aged 18 or older who were regular social media users were invited to take part in an anonymous survey about sexual experiences on social media. As compensation, volunteers were entered into a raffle for a chance to win 50-Euro raffle and received a debrief after the study.

At the beginning of the survey, a screening question was asked: “Have you ever received an unsolicited sexual image?” Those who answered “no” were excluded from the remainder of the survey. Participants who answered “yes” were then given an open-ended question in which they were asked to describe the incident in detail, specifically addressing five aspects: (a) the nature of their relationship with the sender (e.g., stranger, acquaintance, partner); (b) the online platform where the image was received (e.g., Facebook, Instagram, WhatsApp); (c) the situational context (e.g., unsolicited, during a conversation); (d) their emotional reactions (e.g., anger, anxiety); and (e) their behavioral reactions (e.g., ignoring, blocking).

Additionally, sociodemographic information was collected, including gender, age, education level, and sexual orientation. All analyses were conducted using ATLAS.ti version 25.

## Results and Discussion

Most women reported receiving unsolicited genital images from unknown men (55.4%), while 17.4% received them from known men, 14.1% did not specify familiarity with the sender, and 13% had received such images from both known and unknown individuals. These findings support previous research indicating that the majority of unsolicited genital images are sent by strangers (45.7%; Rodríguez-Domínguez et al., 2026), while also pointing to a more complex set of social dynamics in these incidents than previously recognized.

Regarding the platforms through which these images were received, Instagram was reported as the most common social network (44.6%), followed by WhatsApp (18.5%), X (traditionally known as Twitter; 7.6%), Snapchat (6.5%), Facebook (6.5%), Tinder (3.3%), Messenger (3.3%), Omegle (3.3%), other dating apps (2.2%), and Badoo (1.1%). Supporting prior research (Rajan, 2025; UN Women, 2020, 2021), data reflect the wide reach of these incidents across different social media platforms.

Importantly, most participants emphasized that the unsolicited nature of these images—particularly when received in the absence of prior conversation or a sexual context—made them unjustifiable. Specifically, 47.8% reported receiving the image without any prior interaction, 41.3% during conversations that were not sexual in nature and in which they had not expressed any attraction, and 6.5% as a response to previously posted content on their social media. Additionally, 23.9% reported that the unsolicited genital image was accompanied by other forms of cyber-sexual violence, pointing to a pattern of harassment rather than isolated incidents.

In terms of emotional responses, 44.6% of participants reported their emotional reactions. The most frequently reported feelings were rejection or displeasure (58.8%) and disgust (43.1%). Other emotional reactions included fear (11.8%), nervousness or anxiety (11.8%), anger (9.8%), surprise (9.8%), indifference (7.8%), confusion (5.9%), insecurity (3.9%), laughter (3.9%), sadness (2%), and resignation (2%). Notably, 17.6% explicitly described the experience as assaultive and humiliating.

Regarding behavioral responses, a majority of women (67.4%) reported taking no action following the incident. The high rate of inaction may reflect feelings of helplessness, normalization of such behaviors, or uncertainty about how to respond effectively to these incidents (Ringrose et al., 2021; Rodríguez-Domínguez et al., 2026). Others reported blocking the sender (16.3%), confronting the perpetrator (8.7%), reporting the account (4.3%), deleting the image (3.3%), seeking support from friends (3.3%), or adjusting their privacy settings (2.2%).

Overall, these findings underscore the significant reactions to receiving unsolicited genital images and highlight the importance of examining situational and individual factors—including social context and adherence to gender norms—that may influence how women interpret and respond to these experiences. This pilot study was exploratory and preliminary, with a small sample and a nonexperimental design. Its main goal was to gather initial insights and refine the survey and study procedures. Future research is needed to confirm and build upon these early findings.

## Study 2

After identifying emotional reactions to receiving unsolicited sexual images and other situational variables in the pilot study, the main study aimed to examine the role of situational factors and the endorsement of myths about cyber-sexual violence. In this experimental study, we examined how the acceptance of myths about cyber-sexual violence influences women’s evaluations and emotional reactions to unsolicited genital images, depending on situational factors.

Given that the absence of prior interaction and sexual interest was a frequently reported factor in the pilot study, we manipulated the context of a private conversation between a woman and a man on a social media platform. In one scenario (interest condition), both individuals expressed mutual sexual interest. In the other (no interest condition), only the man displayed sexual interest, while the woman did not. Participants were randomly assigned to one of these two scenarios and asked to imagine themselves in the situation. A third group (control condition) viewed a conversation that did not include any instance of incident.

Based on prior research on offline sexual harassment (e.g., Moya-Garófano et al., 2022), we expected that evaluation of the unsolicited genital images in both experimental conditions would be less positive than in the control condition. However, in the interest condition, we hypothesized that the unsolicited image would be evaluated more positively than in the condition where the victim did not express sexual interest (Hypothesis 1).

Moreover, prior studies have indicated that most people perceive unsolicited sexual images as a form of sexual violence (Dietzel, 2022; McArthur et al., 2025). Thus, we anticipated that exposure to an unsolicited genital image in both experimental conditions would increase women’s anxiety (Hypothesis 2a), anger–hostility (Hypothesis 2b), and sadness (Hypothesis 2c), and decrease their happiness (Hypothesis 2d) and feelings of power (Hypothesis 2e), compared to the control condition. We also expected these effects to be stronger in the no interest condition than in the interest condition.

Ringrose et al. (2021) found that some women perceived the receipt of unsolicited genital images as a sign of popularity and desirability, highlighting the role of prior beliefs in shaping the interpretation of these incidents. Therefore, we expected that the evaluation of the unsolicited receipt of a genital image would be moderated by myths about cyber-sexual violence. Specifically, we hypothesized that, after being exposed to a conversation with interest (vs. no interest vs. control condition), women with stronger endorsement of myths about cyber-sexual violence would report more positive evaluation toward the unsolicited receipt of a genital image (Hypothesis 3).

Additionally, drawing from prior literature on offline sexual harassment (e.g., Moya-Garófano et al., 2022), we expected that women’s endorsement of myths about cyber-sexual violence would predict their emotional reactions through their evaluation of the unsolicited genital image. However, we hypothesized that this moderated mediation would occur only in the interest condition, and not in the other two conditions. Specifically, in this condition, women’s endorsement of these myths would be negatively related to anxiety (Hypothesis 4a), anger–hostility (Hypothesis 4b), and sadness (Hypothesis 4c), and positively related to happiness (Hypothesis 4d) and feelings of power (Hypothesis 4e), via more positive evaluations of the situation.

We based these hypotheses on the assumption that women who strongly endorse myths about cyber-sexual violence are more likely to perceive expressions of interest as signals of consent to any sexual interaction, as mentioned above, and thus evaluate and justify the unsolicited receipt of a genital image more positively. This aligns with research showing that such beliefs influence both cognitive appraisals and emotional responses, leading to more favorable evaluations of unsolicited genital images (Newstrom et al., 2021).

### Method

#### Participants

The sample size was estimated prior to data collection using the G*Power 3.1 software. We calculated that a minimum of 159 participants was needed to detect a medium-to-large effect size (f^2^ = 0.25) with a 5% significance level and 80% power in a three-group, between-subjects design. This effect size was selected based on previous research using similar scenario-based designs; for instance, Moya-Garófano et al. (2022) reported medium-to-large effects for participants’ emotional and attitudinal responses to street piropos, which are comparable to the effects we expected to observe in our study. Furthermore, following Simmons et al. (2013), who recommend a minimum of 50 participants per condition to ensure adequate power and reduce false positives, we aimed for at least 150 participants across the three experimental conditions. Additionally, to further assess the power to detect interaction effects involving moderated mediation analyses, a Monte Carlo simulation (Donnelly et al., 2023) was conducted prior to data collection. The simulation modeled three experimental groups, an interaction with a continuous variable, and a continuous mediator influencing the dependent variable. Across 200 simulated datasets, linear models were fitted. The estimated power to detect a small-to-medium interaction effect was approximately 0.80, supporting that a total of 195 participants were necessary for our study.

An initial sample of 323 women was recruited via social media platforms (i.e., Instagram, Facebook, Telegram, WhatsApp, and X) to complete an online survey. In accordance with established guidelines for conducting high-quality research (Goodman & Paolacci, 2017), data from 107 participants were excluded from the analysis: 84 did not complete the survey, 3 did not consent to participate, 3 did not use the social media platforms, 2 reported not having a Spanish nationality, and 15 failed the manipulation check. The final sample consisted of 218 women who were users of social media, with an age range between 18 and 65 years (*M* = 30.35, *SD* = 11.67). On average, they reported using Instagram 1–2 times a day (*M* = 4.28, *SD* = 1.67). Of these, 57.3% of women reported having university education, 24.3% had a general certificate of education, 8.7% had completed vocational training, 6.4% held doctoral studies, 2.3% had secondary studies, and 0.9% held primary education. Among all women, 73.9% identified as heterosexual, 20.2% as bisexual, 4.6% as gay/lesbian, and 1.4% reported other sexual orientations. 

#### Measures and Procedure

Using social media platforms, a message was sent to ask for women volunteers to participate in an online survey on sexual experiences on social media (estimated duration ~ 10 min) and were entered into a 50-Euro raffle as compensation for their participation. The instructions provided to participants clearly stated that they were free to exit the survey at any time without facing any negative consequences. They were reassured that their participation would remain anonymous and that their personal data would be kept confidential. They were also given the contact information of the principal investigator to obtain further details regarding the study's objectives or its outcomes. All analyses were conducted in R.

#### Frequency of Social Network Use

Building upon previous research (Vizcaíno-Cuenca et al., 2025), two items were employed to assess participants’ frequency of social network use. First, participants were asked whether they used social networks (yes/no). Following this, they were asked to specify the amount of time they typically spent on their most frequently used social networks, including WhatsApp, Instagram, Facebook, Twitter (X), Telegram, TikTok, LinkedIn, Badoo, Tinder, Twitch, YouTube, dating websites, and Discord, based on data from international surveys (Kemp, 2025). For the final question, participants rated their usage on a 7-point Likert scale, with responses ranging from 0 (*I don’t use social media*) to 6 (*Continuously throughout the day*).

#### Myths about Cyber-Sexual Violence Against Women

The Acceptance of Myths about Cyber-Sexual Violence Against Women (AMCYS) Scale (Vizcaíno-Cuenca et al., 2025) is a 10-item self-report instrument developed in Spanish to assess participants’ acceptance of myths and beliefs related to cyber-sexual violence. Participants indicate their level of agreement with each statement on a 7-point Likert scale, ranging from 1 (*Totally disagree*) to 7 (*Totally agree*), with higher scores reflecting greater acceptance of myths. An example item is: “Instead of worrying about women who receive a genital image, society should pay more attention to the real victims of sexual assault.” The full set of items in Spanish and English can be found in Vizcaíno-Cuenca et al. (2025). The original validation study demonstrated the scale’s one-dimensionality, which was confirmed in the present sample. The CFA indicated excellent fit (robust ML estimation: RMSEA = 0.050 [90% CI: 0.020, 0.075]; CFI = 0.955; TLI = 0.942; SRMR = 0.044). The scale also showed good internal consistency in our sample (α = .83).

#### The Unsolicited Receipt of a Genital Image

Participants were randomly assigned to one of the three conditions: control (59 women), no interest (89 women), and interest (70 women). Using a hypothetical scenario methodology with a single independent variable (i.e., level of victim sexual interest: no interest vs. interest) where a woman received an unsolicited genital image. Additionally, a control group was exposed to a conversation between a woman and a man without the cyber-sexual violence incident (see each situation in the Online Supplemental Material A). Participants were asked to imagine that they had experienced the conversation that they had been exposed.

#### Manipulation Check

After reading the scenario, participants responded to a manipulation check item (i.e., “Returning to the scenario you previously visualized, please indicate which of the following statements represents the situation: (a) the scenario involves a conversation between two people who did not express sexual interest or exchange sexual images through the chat; (b) the scenario involves a conversation in which only Marcos expressed sexual interest, and additionally, Marcos sent a sexual image to the woman through the chat; (c) the scenario involves a conversation between two people who expressed sexual interest, but only Marcos sent a sexual image to the woman through the chat). Participants who did not select the correct option corresponding to the condition they were assigned were excluded from the analyses, ensuring that only those who accurately understood the scenario were included. This procedure helps maintain the validity of responses to the dependent variables, as participants needed to correctly perceive the scenario to provide meaningful self-reported reactions.

#### Evaluation of the Scenario

Participants responded to seven items to assess participants’ evaluation toward the scenario (Moya-Garófano et al., 2022). The response format was a 7-point Likert scale ranging from 1 (*Not at all*) to 7 (*Very much*). The items included were: fun, pleasant, flattering, chauvinistic, offensive, unpleasant, and (induces) disgust. After reversing the scores for the last four items, an index was calculated by averaging the scores for all items (α = .91 for this study’s sample). Higher scores indicated a more positive attitude toward the scenario.

#### Emotions

Participants answered 12 items of the Scale for Mood Assessment (EVEA; Sanz et al. (2014)) adapted by Moya-Garófano et al. (2022), immediately after reading the scenario. Our objective was to determine the mood participants believed they would experience if confronted with a situation akin to the one described. The selected EVEA subscales assess three distinct moods, each of which is assessed via four items: anxiety (nervous, tense, anxious, restless); anger-hostility (irritated, angry, annoyed, displeased, with rage, outraged, insulted, offended, and humiliated); sadness (melancholic, downcast, and sad); and happiness (happy, optimistic, joyful, cheerful). The response format ranges from 0 (*Not at all*) to 10 (*Very much*). Mean scores were calculated, with higher scores indicating greater levels of anxiety, anger-hostility, sadness, and happiness. In our study, the reliability coefficients were .92 for anxiety, .98 for anger-hostility, .81 for sadness, and .93 for happiness.

#### Feelings of Power

Participants responded to 8 items from Estevan-Reina et al. (2021) to assess the feelings of power and helplessness experienced by women when confronted with the situation described earlier in the unsolicited receipt of genital image conditions (or how they felt after reading the conversation in the control group). The power items included statements such as: I would feel powerful, full of energy, stimulated, and in control of the situation. Conversely, the helplessness items included: I would feel weak, inferior, not in control of the situation, and defenseless. Responses to these items were rated on a 10-point scale, ranging from 0 (*Not at all*) to 10 (*Very much*). In the original validation study, a parallel analysis followed by exploratory factor analysis initially suggested two factors, but the second factor consisted solely of reversed items, indicating an artifact of item wording. A single-factor solution was supported, with all items loading above .54, confirming the scale’s unidimensional structure (Estevan-Reina et al., 2021; Moya-Garófano et al., 2022). In the present study, the scale demonstrated good internal consistency in our Spanish sample (α = .84). Scores on the helplessness items were reversed, and a total score was computed across all items, with higher scores indicating stronger feelings of power.

#### Sociodemographic Information

Finally, participants provided sociodemographic data (i.e., age, gender, sexual orientation, level of education, occupation, nationality, and native language).

## Results

### Preliminary Analyses

A total of 131 women (60.1%) reported having experienced the unsolicited receipt of genital images from unknown men (65.6%), male friends (12.2%), partners/ex-partners (6.1%), and from a combination of these sources (16%). Regarding frequency, 62.6% reported experiencing the unsolicited receipt of genital images once or twice, 16% reported it three or four times, and 21.4% reported it five or more times.

To assess the equivalence of myths about cyber-sexual violence across groups prior to the experimental manipulation, we conducted an ANOVA on participants' scores regarding myths about cyber-sexual violence, using the type of situation as the independent variable. Importantly, the results revealed no significant differences in myths about cyber-sexual violence between the experimental conditions (control condition: *M* = 2.95, *SD* = 1.15; no interest condition: *M* = 2.89, *SD* = 1.22; interest condition: *M* = 2.72, *SD* = 1.18), *F*(2, 215) = 0.64, *p* = .526.

### Evaluation and Reactions to the Unsolicited Receipt of a Genital Image

We conducted separate one-way ANOVAs of the evaluation of the situation ratings, considering type of situation (condition vs. no interest vs. interest) as the between-subjects factor. For these ANOVAs, the estimation of effect size was calculated using partial eta-squared (*η*_p_^2^ ≥ .01 / .06 / .13 indicate small/medium/large effects; Cohen, 1988). Results indicated a main effect of type of situation on evaluation to the situation, *F*(2, 215) = 405.11, *p* < .001, *η*_p_^2^ = 0.79 (Hypothesis 1). Descriptive statistics are shown in Table 1. Post-hoc Bonferroni-corrected tests revealed that the interest condition was perceived more positively than both the no interest condition (*p* < .001;* d* = 0.73) and the control condition (*p* < .001; *d* = 3.44)—where effect sizes of *d* ≥ .20, .50, and .80 represent small, medium, and large effects, respectively (Cohen, 1988). Additionally, the no interest condition was rated more positively than the control condition (*p* < .001; *d* = 5.60).

**Table 1:** Means (and standard deviations) for evaluation and emotions responses toward the situation in each experimental condition

Variables	Control condition (*N* = 59)		No interest condition (*N* = 89)		Interest condition (*N* = 70)	
	*M*	SD	*M*	SD	*M*	SD
Evaluation (1–7)	5.05	0.70	1.34	0.64	1.95	1.04
Anxiety (1–10)	2.02	1.96	6.07	2.95	5.71	2.68
Anger-Hostility (1–10)	1.55	1.63	7.92	2.24	6.87	2.60
Sadness (1–10)	1.53	1.61	3.85	2.67	3.63	2.49
Happy (1–10)	4.73	2.54	1.27	0.77	1.77	1.57
Feelings of power (1–10)	6.41	1.23	3.56	1.45	3.85	1.62

Control = Control condition with no unsolicited genital image; No interest = Scenario in which the victim did not show interest and received an unsolicited genital image; Interest = Scenario in which the victim showed interest and received an unsolicited genital image

We performed a multivariate analysis of variance (MANOVA) on participants’ reactions (anxiety, anger-hostility, sadness, happiness, and feelings of power) to the situation, with the type of situation (control vs. no interest vs. interest) as a between-subjects factor. Results (Wilks’s lambda = .33, *F*(8, 424) = 39.81, *p* < .001, *η*_p_^2^ = .43) revealed that the manipulation had a significant multivariate effect. As expected, we observed the main effect of the type of situation on anxiety, *F*(2, 215) = 47.77, *p* < .001, *η*_p_^2^ = .31, anger-hostility,* F*(2, 215) = 156.62, *p* < .001, *η*_p_^2^ = .59, sadness, *F*(2, 215) = 19.14, *p* < .001, *η*_p_^2^ = .15, happiness, *F*(2, 215) = 82.99, *p* < .001, *η*_p_^2^ = .44, and feelings of power, *F*(2, 215) = 76.47, *p* < .001, *η*_p_^2^ = .42. Post-hoc Bonferroni tests revealed that women in the control condition reported significantly less anxiety than women in the no interest condition (*p* < .001; *d* = 1.56) and in the interest condition (*p* < .001; *d* = 1.56); anxiety ratings in the other two conditions about the unsolicited receipt of genital images did not differ (*p* = 1.00; *d* = 0.13; Hypothesis 2a). Women in the control condition reported significantly lower anger-hostility than women in the no interest condition (*p* < .001; *d* = 2.87) and in the interest condition (*p* < .001; *d* = 2.40); women in the interest condition also reported lower anger-hostility than those in the no interest condition (*p* = .011; *d* = 0.47) (Hypothesis 2b). Additionally, women in the control condition experienced significantly lower sadness than those in the no interest condition (*p* < .001; *d* = 0.98) and in the interest condition (*p* < .001; *d* = 0.89); however, there were no differences between the no interest and the interest conditions (*p* = 1.00; *d* = 0.09; Hypothesis 2c). Similarly, happiness scores were higher in the control condition compared to the no interest condition (*p* < .001; *d* = 2.08) and the interest condition (*p* < .001; *d* = 1.78). However, no differences were found between the no interest condition and the interest condition (*p* = .191; *d* = 0.30; Hypothesis 2d). Finally, in the control condition, feelings of power were higher than in the no interest condition (*p* < .001; *d* = 1.96) and the interest condition (*p* < .001; *d* = 1.36); women reported similar feelings of power in the no interest condition and the interest condition (*p* = .625; *d* = 0.20; Hypothesis 2e).

Table 2 shows the correlations among the variables for the unsolicited receipt of genital images and the control condition.

**Table 2:** Descriptive statistics for all scales and Pearson correlations

	Unsolicited receipt of genital images conditions		Control condition		(1)	(2)	(3)	(4)	(5)	(6)	(7)	(8)
	*M*	SD	*M*	SD								
(1) Age (18–65)	30.00	11.27	31.31	12.73	–	.12	−.07	−.16	−.09	−.09	−.06	.07
(2) AMCYS (1–7)	2.82	1.21	2.95	1.15	−.06	–	−.09	−.05	.02	.01	−.12	−.09
(3) Evaluation (1–7)	1.61	0.89	5.05	0.70	.04	.26**	–	−.53***	−.67***	−.64***	.51***	.67***
(4) Anxiety (0–10)	5.91	2.83	2.02	1.96	−.06	−.21**	−.36***	–	.86***	.79***	−.12	−.46***
(5) Anger-Hostility (0–10)	7.46	2.45	1.55	1.63	−.09	−.14	−.61***	.76***	–	.98***	−.12	−.54***
(6) Sadness (0–10)	3.76	2.59	1.53	1.61	−.07	−.10	−.26***	.71***	.62***	–	−.06	−.53***
(7) Happy (0–10)	1.49	1.21	4.73	2.54	.19*	.19*	.51***	−.23**	−.43***	−.14	–	.57***
(8) Feelings of Power (0–10)	3.69	1.53	6.41	1.23	.21**	.22**	.54***	−.71***	−.74***	−.62***	.44***	–

Unsolicited receipt of genital images Conditions = Conditions in which the victims (interest and no interest) received an unsolicited genital image; Control = Control condition with no unsolicited genital image. Unsolicited receipt of genital images conditions correlations below diagonal (*N* = 159); Control condition correlations above diagonal (*N* = 59). * *p* < .05; ** *p* < .01; *** *p* < .001

As shown in Table 2, age correlated positively with happiness (*r* = .19, *p* = .015) and feelings of power (*r* = .21, *p* = .008) in the unsolicited receipt of genital images condition. However, the correlations between age and the other variables were not significant in both conditions (*p*_s_ > .22).

Higher levels of myths about cyber-sexual violence were associated with a more favorable evaluation of the unsolicited receipt of a genital image (*r* = .26, *p* = .001), but not with the evaluation in the control condition (*r* = -.09, *p* = .510). A more positive evaluation of both situations (control and unsolicited genital image) was negatively correlated with anxiety, anger-hostility, and sadness, and positively correlated with feelings of happiness and power (*p*_s_ < .05).

### Myths about Cyber-Sexual Violence and Reactions to the Unsolicited Receipt of a Genital Image

Using the PROCESS macro (Hayes & Preacher, 2013), we conducted five moderated mediation analyses (Model 8), one for each emotional reaction (i.e., anxiety, anger-hostility, sadness, happiness, and feelings of power), with 10,000 bootstrap resamples. For analytic clarity, and following the approach of Moya-Garófano et al. (2022), myths about cyber-sexual violence were introduced as an antecedent variable, evaluation of the situation as a mediator variable and experimental manipulation as a moderator variable. We included the extent to which they have imagined the situation and the previous experience of receiving unsolicited genital images as covariates. Experimental conditions were represented in two orthogonal contrasts. The first contrast, C1, compared the no interest condition, coded as 1, with the other conditions (i.e., control and interest), coded as 0. The second contrast, C2, compared the interest condition, coded as 1, with the other conditions (i.e., control and no sexual interest), coded as 0. We calculated the standardized effect size f^2^ for the interaction between myths about cyber-sexual violence and experimental manipulation (f^2^ ≥ .02/ .15/ .35 indicate small/medium/large effects; Cohen, 1988) based on the change in R^2^ (Δf^2^). In the presence of a significant interaction, we selected simple slope analyses to test the effect of type of situation at high (84th) and low (16th) values of myths about cyber-sexual violence. Following the recommendations of Hayes and Rockwood (2017), we did not include values that were out the range of the data sample and therefore selected percentiles as conditioning values (i.e., above the maximum or below the minimum of observed values). All results are shown in Table 3.

**Table 3:** Moderated mediation analyses

	Evaluation of the situation			Anxiety			Anger-Hostility			Sadness			Happy			Feelings of power		
	*Β*	SE	95% CI	*Β*	SE	95% CI	*Β*	SE	95% CI	*Β*	SE	95% CI	*Β*	SE	95% CI	*Β*	SE	95% CI
AMCYS	−.08	.09	[−.25, .10]	−.15	.28	[−.70, .40]	−.07	.20	[−.47, .33]	−.01	.26	[−.52, .50]	−.25	.17	[−.59, .09]	−.06	.14	[−.33, .21]
Evaluation	–	–	–	−1.22	.22	[−1.65, −.79]	−1.69	.16	[−2.01, −1.38]	−.90	.20	[−1.30, −.50]	.90	.14	[.63, 1.16]	.99	.11	[.78, 1.20]
C1	−3.73	.13	[−3.99, −3.48]	−.48	.91	[−2.27, 1.31]	.08	.67	[−1.23, 1.39]	−1.01	.84	[−2.67, .66]	−.15	.56	[−1.26, .96]	.81	.45	[−.07, 1.70]
C2	−3.11	.14	[−3.38, −2.84]	−.09	.80	[−1.67, 1.50]	.12	.59	[−1.04, 1.28]	−.61	.75	[−2.08, .87]	−.22	.50	[−1.21, .76]	.47	.40	[−.32, 1.25]
AMCYS x C1	.21	.12	[−.01, .43]	−.17	.36	[−.87, .53]	.02	.26	[−.50, .53]	−.02	.33	[−.67, .63]	.34	.22	[−.10, .77]	.20	.18	[−.15, .55]
AMCYS x C2	.44	.12	[.21, .68]	.09	.39	[−.67, .86]	.30	.29	[−.26, .86]	−.03	.36	[−.75, .68]	.25	.24	[−.23, .72]	.07	.19	[−.31, .44]
*Conditional Direct Effects*																		
Conditions																		
Control	−.08	.09	[−.25, .10]	–	–	–	–	–	–	–	–	–	–	–	–	–	–	–
Interest	.13	.07	[.00, .26]															
Victim interest	.36	.08	[.21, .52]	–	–	–	–	–	–	–	–	–	–	–	–	–	–	–
*Conditional Indirect Effects*																		
Conditions																		
Control	–	–	–	.10	.09	[−.08, .28]	.13	.12	[−.11, .37]	.07	.07	[−.07, .21]	−.07	.07	[−.21, .05]	−.08	.07	[−.22, .06]
No interest	–	–	–	−.16	.07	[−.30, −.03]	−.22	.10	[−.42, −.04]	−.12	.05	[−.23, −.02]	.12	.06	[−.02, .24]	.13	.06	[.02, .25]
Interest	–	–	–	−.44	.22	[−.88, −.03]	−.62	.29	[−1.12, −.05]	−.33	.17	[−.66, −.02]	.33	.18	[.02, .69]	.36	.18	[.03, .69]

Bootstrap sample size = 10,000. IC: Confidence Interval. Contrast C1 compared the no interest condition with both the control and interest conditions, whereas contrast C2 compared the interest condition with both the control and no interest conditions

Following our theoretical perspective and for transparency, we also conducted supplementary analyses reversing the roles of the variables—introducing experimental condition as the antecedent variable and myths about cyber-sexual violence as the moderator. These supplementary results are presented in Online Supplementary Material B.

**Fig. 1:**
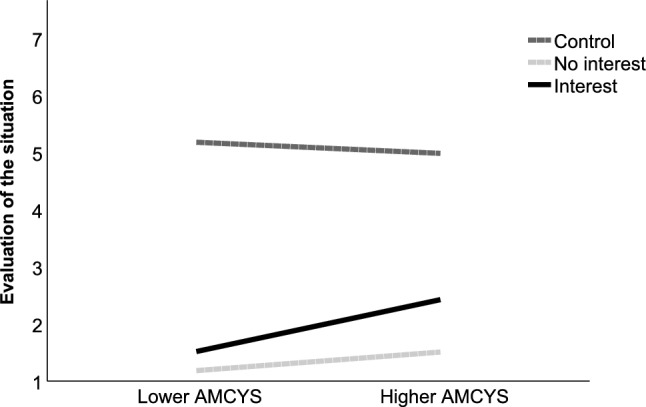
Evaluation of the situation as a function of AMCYS and type of situation. Note. The impact of participants lower (16th) and higher (84th) in AMCYS scores on evaluation of the situation as a function of the type of situation (control vs. no interest vs. interest). Solid lines indicate significant relationship.

First, results did not show a main effect of the acceptance of myths about cyber-sexual violence on the evaluation of the situation (*b* = -0.08, *SE* = 0.09, *t* = -0.89, *p* = .377, 95% CI [-0.25, 0.10]). Nevertheless, as shown in Fig. 1, the model showed an interaction between myths about cyber-sexual violence and type of situation on evaluation of the situation (Hypothesis 3; Δ*f*^2^ = .06). There was no interaction between the first contrast (C1) and myths about cyber-sexual violence (*b* = 0.21, *SE* = 0.11, *t* = 1.86, *p* = .064, 95% CI [-0.01, 0.43]); however, the interaction between the second contrast (C2) and myths about cyber-sexual violence (*b* = 0.44, *SE* = 0.12, *t* = 3.68, *p* < .001, 95% CI [0.21, 0.68]) was statistically significant. Women with higher myths about cyber-sexual violence reported more positively evaluation of the unsolicited receipt of a genital image in the interest condition (*b* = 0.36, *SE* = 0.08, *t* = 4.57, *p* < .001, 95% CI [0.21, 0.52]). However, this relationship was not statistically significant in the no interest condition (*b* = 0.13, *SE* = 0.07, *t* = 1.93, *p* = .055, 95% CI [0.00, 0.26]) and in the control condition (*b* = -0.08, *SE* = 0.09, *t* = -0.89, *p* = .377, 95% CI [-0.25, 0.10]).

**Fig. 2:**
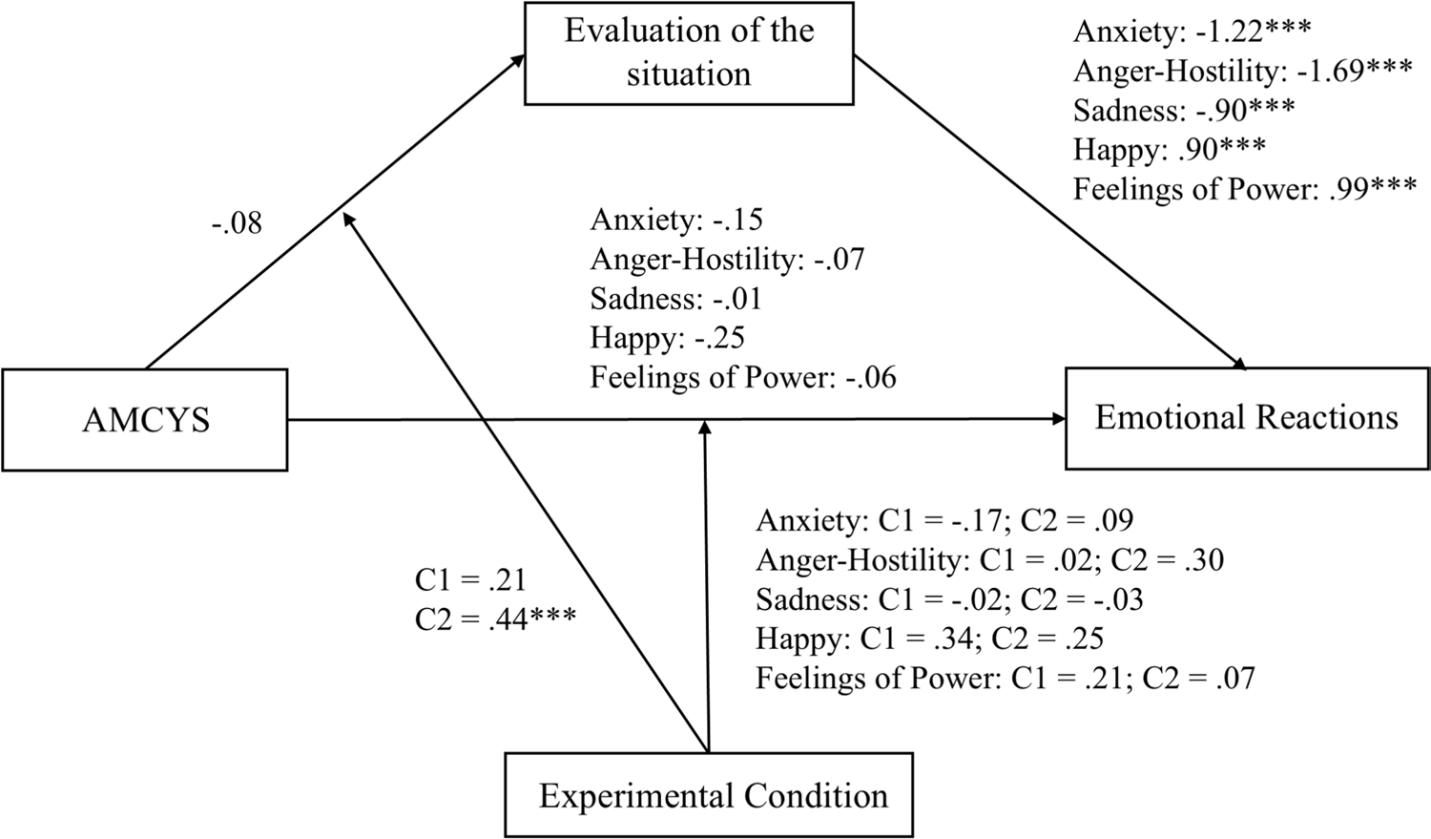
Moderated mediation model proposed. *Note N* = 218. *p < .05. **p < .01. ***p < .001. Contrast C1 compared the no interest condition with both the control and interest conditions, whereas contrast C2 compared the interest condition with both the control and no interest conditions.

Importantly, the results indicated that the moderated mediation analyses were significant across the five dependent variables (see Table 3; also see Fig. 2). Regarding hypotheses 4a–4e, the indirect effect of AMCYS on emotions through evaluation of the situation was only significant in the interest condition: indices for anxiety (Index = -0.54, *SE* = 0.25, 95% CI [-1.02, -0.07]; Hypothesis 4a), anger-hostility (Index = -0.75, *SE* = 0.32, 95% CI [-1.33, -0.10]; Hypothesis 4b), sadness (Index = -0.40, *SE* = 0.19, 95% CI [-0.77, -0.05]; Hypothesis 4c), happiness (Index = 0.40, *SE* = 0.21, 95% CI [0.05, 0.81]; Hypothesis 4d), and feelings of power (Index = 0.44, *SE* = 0.20, 95% CI [0.06, 0.81]; Hypothesis 4e) were all significant. As shown in Fig. 2, women who scored higher in myths about cyber-sexual violence held more positive evaluation of the unsolicited receipt of a genital image in the interest condition, which in turn was associated with lower anxiety, anger-hostility, and sadness, as well as greater happiness and feelings of power. The direct effects (see Table 3 and Fig. 2) did not reveal any significant relationship between myths about cyber-sexual violence and emotional reactions to the unsolicited receipt of a genital image in either the interest or no interest conditions, compared to the control condition.

## Discussion

The findings highlight that women’s emotional and evaluative responses to the unsolicited receipt of genital images are primarily negative. However, their evaluations and emotional reactions can vary significantly depending on the perceived context of the interaction and their prior beliefs. Situations framed as involving sexual interest from the woman were evaluated more positively compared to situations where there was no sexual interest and where there was no incident (i.e., receiving an unsolicited genital image); however, the sexual context did not affect directly the emotional reactions. Importantly, only women who endorsed more (vs. fewer) myths about cyber-sexual violence were more likely to evaluate such situations positively in the interest condition (vs. no interest and control conditions), and this evaluation was associated with a higher proportion of positive emotions and a lower proportion of negative ones. These results are in line with prior research (i.e., Moya-Garófano et al., 2022) and suggest that social norms and myths about cyber-sexual violence shape how women respond to such incidents.

## General Discussion

Unsolicited genital images, despite their frequent occurrence and the accumulating negative impacts on women (Iroegbu et al., 2024; Vizcaíno-Cuenca et al., 2026), are often downplayed and questioned as a form of violence, with their consequences trivialized (Hayes & Dragiewicz, 2018; Vizcaíno-Cuenca et al., 2024). Our research highlights that women generally evaluated the unsolicited receipt of genital images negatively (Hypothesis 1), experiencing increased anxiety, anger, hostility, and sadness, alongside decreased happiness and feelings of power, regardless of whether there was a preexisting sexual interest context (Hypotheses 2a-2e). However, the influence of myths about cyber-sexual violence shaped how women evaluated and emotionally responded to these incidents. Specifically, we found that women who more strongly endorsed such myths were more likely to evaluate unsolicited genital images positively (Hypothesis 3). This positive evaluation, in turn, was associated with reduced anxiety, anger-hostility, and sadness, and with increased happiness and feelings of power—particularly when the woman had previously shown sexual interest (vs. no interest vs. control condition; Hypotheses 4a-4e).

It is important to emphasize that, overall, women did not enjoy receiving unsolicited genital images, and the immediate and cumulative emotional harm caused by such experiences may have important emotional impacts. Supporting our findings, qualitative studies have shown that women often react with fear, anxiety, and disgust to unsolicited sexual images (Jeacock et al., 2025), frequently responding to the perpetrator with rejection or by not responding at all (Rodríguez-Domínguez et al., 2026). Our results further demonstrated that women experienced more negative and fewer positive emotional reactions to unsolicited genital images, even when the scenario involved a preexisting sexual interest context—showing no significant difference from situations where the victim did not show sexual interest. These findings challenge social narratives that justify the unsolicited sending of genital images by referencing the victim’s behavior, suggesting the act is consensual or even desired (Centelles et al., 2021; Vizcaíno-Cuenca et al., 2024). Our data and prior literature underscore that expressions of desire or interest, or even the existence of a romantic relationship with the perpetrator (e.g., Marcotte et al., 2021), do not imply that women welcome or enjoy experiencing unwanted sexual attention (e.g., unsolicited genital images).

Our research also showed that the evaluation of the unsolicited genital image was correlated with the emotional reactions they triggered. Specifically, in both experimental conditions (i.e., interest and no interest), women who evaluated less positively the unsolicited genital images reported lower happiness and feelings of power, and greater anxiety, anger, and sadness. This is in line with prior research on street harassment which highlighted that the evaluation or perception of the incident may affect the emotional reactions (Moya-Garófano et al., 2022) and, even so, behavioral reactions (e.g., direct confrontation, deleting social networking; Rodríguez-Domínguez et al., 2026).

However, our findings also highlight that both the evaluation and emotional responses were not only influenced by the unsolicited genital image itself, but also by individual and situational factors. Previous research has shown that a few women interpreted these images as a sign of popularity or desirability (Ringrose et al., 2021), perceived them as flattering, assumed perpetrators were sent with good intentions, or dismissed them as a joke (Rodríguez-Domínguez et al., 2026). However, our results suggest that such evaluations may be shaped by beliefs that normalize these incidents. Specifically, we found that women who endorsed more myths about cyber-sexual violence evaluated the unsolicited genital image more positively (compared to the control condition without an incident), which in turn was associated with greater feelings of happiness and power, and lower levels of anxiety, anger, and sadness. Notably, this pattern emerged only when the scenario involved a preexisting victim’s sexual interest, and not when the victim did not show sexual interest.

The specialized literature on offline settings suggests that the acceptance of rape myths helps construct a “victim prototype” and defines which behaviors are classified as harmful versus those normalized as expressions of sexual desire (Bohner et al., 2023). Consequently, these beliefs may be associated with a short-term emotional buffering effect for some women in response to unsolicited sexual images. Supporting this, Bohner et al. (1993) found that women who had not been raped and rejected rape myths reported lower positive feelings and self-esteem after reading a rape testimony, compared to women who had not been raped but endorsed stronger rape myths. Regarding subtle forms of sexual harassment, such as cat-calling, Moya–Garófano et al. (2022) found that women who endorsed more benevolent sexism evaluated street harassment more positively, which in turn was associated with more positive and fewer negative feelings. Our study aligns with these results, suggesting that women with stronger myths about cyber-sexual violence are more likely to trivialize the unsolicited receipt of genital images, particularly when there is a prior sexual interest from the victim. Attribution theory (Heider, 1958) offers a useful framework for understanding this pattern.

According to attribution theory, individuals draw on prior beliefs to interpret ambiguous or unexpected situations. In the context of unsolicited genital images, women may rely on preexisting beliefs—such as myths about cyber-sexual violence—to make sense of these experiences, particularly when incidents occur following prior exchanges of messages or based on cues from their online profiles (Centelles et al., 2021). Our findings suggest that endorsement of such beliefs shapes how these situations are evaluated and, in turn, are associated with more positive and fewer negative emotional responses, consistent with previous research (Bohner et al., 2023).

However, prior research indicates that, in broader social contexts, these beliefs can be linked to potential long-term negative outcomes among women who experience these incidents frequently (Vizcaíno-Cuenca et al., 2026), underscoring the need to understand under what conditions short-term protective effects persist or may eventually give way to harm. In addition to the influence of myths about cyber-sexual violence, other factors may help explain why some women experience less immediate distress, including repeated exposure, social expectations—both the behaviors women observe others doing and the behaviors they feel they should follow—(Rodríguez-Domínguez et al., 2026), the tendency to minimize reactions to protect the sender or avoid social conflict, and awareness of acceptance of double sexual standard (Ringrose et al., 2021). Together, these findings suggest that emotional responses to unsolicited sexual images are shaped by multiple, interacting social and individual processes, highlighting the complexity of cyber-sexual violence and the need for continued research.

### Theoretical and Practical Implications

Our study has important implications. From a theoretical perspective, our research highlights that, although unsolicited genital images are generally perceived as unpleasant and unwanted by women, myths about cyber-sexual violence can shape how these incidents are evaluated and how individuals emotionally respond to them. These myths—rooted in what Russell (1975) termed mystic femininity—promote the idea that women are expected to present themselves as sexually available or receptive in online interactions. When these myths intersect with preexisting and often distorted beliefs about implicit sexual consent, they may contribute to the normalization or misinterpretation of harmful behaviors (Bohner et al., 2023; Moya-Garófano et al., 2022). As a result, behaviors such as unsolicited genital images may not be immediately recognized as violent or inappropriate but instead may be mistakenly perceived as expressions of interest or desirability, reinforcing traditional gender roles that associate femininity with passive sexual acceptance (Ringrose et al., 2021). Importantly, our results show that women who more strongly endorsed these myths reacted less negatively to such incidents. This does not mean the behaviors are harmless; rather, our findings suggest they reflect internalized sexist norms that foster victim self-blame, hinder recognition of abuse, and help trivialize its consequences (i.e., Bohner et al., 2023; Durán & Rodríguez-Domínguez, 2023; Moya-Garófano et al., 2022).

From a practical perspective, our findings underscore the importance of acknowledging the emotional and cognitive consequences associated with repeated exposure to unsolicited genital images (e.g., Iroegbu et al., 2024; Vizcaíno-Cuenca et al., 2026). While endorsement of myths may buffer immediate emotional responses, evidence suggests that long-term exposure can have detrimental effects (Rodríguez-Domínguez et al., 2026; Vizcaíno-Cuenca et al., 2026). Therefore, interventions should aim to challenge these myths—both among women and the broader population—so that women do not feel compelled to conform to a (cyber)rape culture that, in the long run, restricts and harms not only them but individuals of all genders.

### Limitations and Future Research

This study is not without limitations. Firstly, the unsolicited receipt of genital images was examined exclusively within a heteronormative and binary framework. However, research suggests that sexual minorities also receive unsolicited genital images, and that sexist ideologies may influence how these experiences are evaluated and emotionally processed. In addition to expanding beyond heteronormative frameworks, future research should examine multiple predictors of participants’ responses—including beliefs in cyber-sexual violence myths, previous experiences of cyber- or face-to-face harassment, hostile or benevolent sexism, as well as potentially protective or empowering factors such as feminist beliefs, attitudes toward sexuality and pornography, enjoyment of self-sexualization, and levels of self-determination or agency. Examining these predictors together could clarify why participants’ emotional and behavioral responses vary and help disentangle the influence of harmful beliefs from that of protective or empowering factors.

Secondly, while this phenomenon has been reported across various countries, our findings are specific to Spain. Data were collected from convenience samples of self-identified women social media users in Spain, and participants of non-Spanish nationality were excluded to reduce cultural bias and ensure measurement validity (Goodman & Paolacci, 2017; Qureshi et al., 2009). Given that our instruments were adapted for the Spanish context, this restriction was necessary. Nevertheless, replicating these studies in other countries with culturally adapted measures is essential to assess how local norms and endorsement of gender roles may influence the phenomenon and to determine the generalizability of our results.

Third, the study employed a scenario-based methodology, which, while useful for evaluating the influence of theoretical or contextual factors on individuals’ attitudes—and has been shown to closely approximate real-world responses (Hainmueller et al., 2015)—cannot fully replicate the actual experiences of receiving unsolicited genital images that women encounter daily in online spaces. The self-reported feelings elicited in this scenario are hypothetical and should not be interpreted as direct measures of real-world emotional consequences or mental health impacts; rather, they provide insight into potential responses under controlled conditions. Future research could incorporate virtual reality or other immersive methods to better simulate these situations, also taking into account additional situational factors, such as whether the participant had previously posted an image on her profile. Moreover, since the design was based on a single cross-sectional experiment with a convenience sample, causal conclusions cannot be drawn; longitudinal research is needed to examine how these mechanisms develop over time.

Finally, it would be valuable to investigate how myths about cyber-sexual violence influence not only emotional reactions but also behavioral responses, such as confrontation, blocking, deleting accounts, or reporting to authorities. Expanding experimental designs to include these behavioral outcomes would provide a more comprehensive understanding of participants’ responses to unsolicited genital images.

### Conclusions

Our study contributes to the growing literature on the unsolicited receipt of genital images, a widespread phenomenon that affects women globally. The core message of our findings is that women generally experience such incidents as aversive—even in situations where they feel attracted to the perpetrator. However, endorsement of myths about cyber-sexual violence was associated with some women perceiving these incidents more positively and reporting stronger positive and weaker negative emotions in situations in which they showed interest in the perpetrator*.* Crucially, these seemingly more benign emotional responses do not indicate that the behavior is harmless or acceptable. Rather, they may reflect internalized narratives that obscure the coercive and violent nature of the act—or even give rise to feelings of self-blame for having believed they provoked such a response from the perpetrator—ultimately trivializing its consequences (e.g., Bernstein et al., 2024; Bohner et al., 2023). In this sense, the endorsement of such myths may operate as a psychological mechanism that hinders recognition of the experience as harmful experiences, reinforcing harmful norms and potentially increasing vulnerability to future victimization.
